# Identification of clinical diagnostic and immune cell infiltration characteristics of acute myocardial infarction with machine learning approach

**DOI:** 10.1038/s41598-025-11957-0

**Published:** 2025-07-20

**Authors:** Huali Jiang, Weijie Chen, Benfa Chen, Tao Feng, Heng Li, Dan Li, Shanhua Wang, Weijie Li

**Affiliations:** 1 Department of Cardiovascularology, Dongguan Key Laboratory of Prevention and Treatment for Chronic Cardiovascular Diseases, Dongguan Tungwah Hospital, Dongguan, China; 2 Department of Cardiovascularology, Dongguan Songshan Lake Tungwah Hospital, Dongguan, China; 3 Department of Cardiology, Zhongshan People’s Hospital, Zhongshan, China; 4 Department of Cardiovascularology, The Sixth People’s Hospital of Huizhou City, Huiyang Hospital Affiliated to Southern Medical University, Huizhou, China

**Keywords:** Acute myocardial infarction, Immune cell infiltration, Machine learning, Diagnosis, Bioinformatics, Computational biology and bioinformatics, Biomarkers

## Abstract

Acute myocardial infarction (AMI) is a serious heart disease with high fatality rates. The progress of AMI involves immune cell infiltration. However, suitable clinical diagnostic biomarkers and the roles of immune cells in AMI remain unknown. Three datasets (GSE61145, GSE34198, and GSE66360) were used from Gene Expression Omnibus. Dysregulated expression of genes was screened and functionally analyzed. Weighted Gene Co-expression Network Analysis (WGCNA) was used to identify significant module genes associated with AMI. Machine learning algorithms (Support Vector Machine (SVM), Random Forest (RF) and Least Absolute Shrinkage and Selection Operator (LASSO)) were applied to identify hub genes. Subsequently, receiver operating characteristic curves (ROC) were generated to evaluate the risk of AMI patients. Finally, immune cell infiltration were assessed by CIBERSORT, correlation analysis and immunohistochemistry. A total of 134 upregulated and 25 downregulated genes were identified. Functional analysis showed that the dysregulated genes were involved in cytokine- and immune-related signaling. Ten hub genes were used to establish a diagnostic model. Immune cell infiltration analysis showed that ten genes were correlated with activation of various immune cells; specifically, naive B cells, activated CD4 memory T cells, and resting mast cells were significantly associated with AMI. Immunohistochemical staining indicated that *FOS* and *IL18RAP* were significantly upregulated in AMI, CD4 naive T and neutrophils were significantly infiltrated in the microenvironment of AMI. The hub genes involved in activating immune cell infiltration and developing AMI could act as promising diagnostic biomarkers and targets for clinical treatment of AMI.

**Supplementary Information:**

The online version contains supplementary material available at 10.1038/s41598-025-11957-0.

## Introduction

Acute myocardial infarction (AMI) is a deleterious coronary heart disease that decreases blood supply to the coronary artery^1^. AMI is the leading cause of death and disability, specifically in middle-aged individuals^2^. Inflammatory and immune regulators cause irreparable damage to the myocardial cells, which cause detrimental complications, such as malignant arrhythmia, and even heart failure^3^. Therefore, early diagnosis and timely treatment of AMI are of profound significance to prevent disease progression.

Cardiac troponins (cTns) are extremely important diagnostic markers for cardiovascular diseases including AMI^4^. However, cTns are released only after myocardial injury, and therefore, AMI cannot be early diagnosed^5^. Meanwhile, in clinical application, false-positive cases of increased cTns also lead to misdiagnosis and even wrong treatment, which causes serious consequences to the patient^6^. Therefore, identifying a promising biomarker to predict and treat AMI is urgent. Transcriptome and expression profile sequencing have been widely used in AMI studies^7^. However, data from a single center or adopting a single approach may not be adequate in identifying biological markers of AMI.

The majority of available target gene screening approaches rely on transcriptomic analysis, principal component analysis, or association^8^. Evidence suggests that analyzing significance cannot explain a critical fraction of phenotypic variation, and this technique has resulted in a limited capacity to find high-risk populations at rigorous significance levels. Weighted gene co-expression network analysis (WGCNA) is a classic bioinformatics approach that may be used to assess various gene expression profiles and investigate their association with clinical characteristics^9^. Compared to traditional approaches, machine learning, a prototype after a systematic search for high-dimensional collection various predictors, might be a beneficial tool for improving clinical encounters for disease detection and prevention^10^.

In this study, we identified differential expression analysis, enrichment analysis, and investigated candidate hub genes of AMI by integrating the Gene Expression Omnibus (GEO) database with Weighted Gene Co-expression Network Analysis (WGCNA) and three machine learning algorithms, including Support Vector Machine (SVM), Random Forest (RF) and Least Absolute Shrinkage and Selection Operator (LASSO). Immune infiltration analysis and *vivo* experiments identified that *FOS* and *IL18RAP* were associated with the infiltration of CD4 naive T and neutrophils in AMI, which provide new insights for the diagnosis and treatment of clinicians.

## Materials and methods

### Data acquisition and processing

RNA expression profiles in AMI (GSE61145, GSE34198, and GSE66360) were obtained from the Gene Expression Omnibus (GEO) database (https://www.ncbi.nlm.nih.gov/geo/). The clinical details of GEO datasets are listed in Table S1. For data prepossessing, probes were mapped to genes and empty probes were removed. Multiple probes corresponded to the same gene, and the median value was chosen as the expression of the gene. The batch effect from the data in GSE61145 was removed from the combat function in the SVA package of R software^11^.

### Identification of differentially expressed genes (DEGs)

Background correction, normalization and gene symbol conversion were performed on the integrated AMI datasets (GSE61145 (*n* = 57; GPL6106, GPL6884), GSE34198 (*n* = 97; GPL6102), and GSE66360 (*n* = 99; GPL570)). The dysregulated expression analysis was identified using the R package “limma”^12^ and heatmap and volcano plots were constructed. Therefore, DEGs in AMI dataset were screened upon the thresholds of adjusted *p* ≤ 0.05 and |log2 (fold change)| ≥ 0.585. Subsequently, the expression patterns of DEGs were visualized in the form of volcano plots and heatmaps with the “ggplot2” package and “pheatmap” package in R software, respectively.

### Function enrichment analysis

As the method used in our previous study^13^, the Gene Ontology (GO) annotation and Kyoto Encyclopedia of Genes and Genomes (KEGG) pathway enrichment analysis were performed using the clusterProfiler package of R software. During Gene Ontology analysis, biological process (BP), cellular component (CC) and molecular function (MF) were identified. The top 10 GO terms were visualized using the R package “ggplot2” in each category. False discovery rate < 0.05 and *p* < 0.05 were considered as significantly enriched functions and pathways.

### Weighted gene co-expression network analysis (WGCNA) and key module genes identification

WGCNA was used as a systematic biological strategy to discover gene association patterns across multiple samples and to identify possible biomarker genes or therapeutic targets based on gene set interconnectivity and phenotypic correlation. First, the median absolute deviation of each gene in the AMI datasets was calculated, then genes with median absolute deviation of 0 were removed from each sample. Second, the “goodSamplesGenes” function of the “WGCNA” package was used to evaluate the unqualified genes and samples^14^. Third, the one-step network construction function of the “WGCNA” package was used to construct a scale-free co-expression gene network. Meanwhile, the soft threshold power (β = 10) was taken as the weight value with the following parameters: powerVector = c(c(1:10), seq(from = 12, to = 30, by = 2)), networkType = “unsigned”, and verbose = 5. Then, the blockwise Modules function was used to build the WGCNA network, and the key parameters were as follows: power = ‘8’, max Block Size = ‘6343’, topological overlap matrix (TOM) Type = ‘unsigned’, min Module Size = ‘30’, reassign Threshold = ‘0’, merge Cut Height = ‘0.25’, numeric Labels = ‘TRUE’, pam Respects Dendro = ‘FALSE’, save TOMs= ‘TRUE’, cor Type = ‘pearson’, Max POutliers= ‘1’, load TOMs= ‘TRUE’, and verbose = ‘3’. Fourth, after obtaining the modules, the different module eigengenes were obtained based on the first principal component of the module expression, while the module-trait relationships were evaluated in line with the association between module eigengenes and clinical characteristics. Fifth, the modules with the most significant positive and negative correlations of module-trait relationships were screened. Then, module membership and gene significance scores were also assessed thesignificance of modules .

### Machine learning

To identify the hub genes and establish a diagnostic model of AMI, Support Vector Machine (SVM) analysis was conducted using the R software caret package^15^. SVM linear method was used to screen hub genes by 5-fold cross validation. Random Forest (RF) was analyzed using the RF function in R software RF package^16^. First, the values of the parameters mtry (the number of variables used in the binary tree in the specified node) and nTree (the number of decision trees contained in the RF) in the model were determined; then the RF model was constructed; and the top 30 genes were displayed according to mean decrease accuracy and mean decrease gini. The Least Absolute Shrinkage and Selection Operator (LASSO) algorithm, a logistic regression method for filtering variables to enhance the predictive performance, was initially adopted in this work to screen the candidate biomarkers with the “glmnet” package^17^. The concrete details were listed as follows: nfolds = ‘5’, family = ‘binomial’, and type measure = ‘deviance’. The hub genes were screened under 5-fold cross validation. Based on WGCNA, SVM, RF and LASSO, the selected markers are taken from at least 19 genes that exist in at least 2 methods. Then, the stepwise regression method is used to further reduce the gene set, and finally 10 genes are obtained to establish a diagnostic model.

### Receiver operating characteristic (ROC) curve analysis

For the GSE34198 and GSE66360 datasets, ROC curves were constructed based on the expression of ten differential hub genes. Receiver operating characteristic (ROC) curves and the area under the ROC curves (AUC) were performed to evaluate the diagnostic accuracy of the hub genes using SigmaPlot 12.5 software (Systat Software, Inc., San Jose, CA, United States).

### Immune cell infiltration analysis

To evaluate the characteristics of infiltrating immune cells in healthy control and AMI patients, the GEO datasets were prepared and 22 immune cell subpopulations were defined by the CIBERSORT gene signature file LM22^18^. The expression data were imported into CIBERSORT using R and then iterated 1000 times to estimate the relative proportion of each immune cell type.

### Establishment of myocardial infarction model in vivo

The AMI model was developed as previously mentioned^19^. After 12 h of myocardial infarction, C57BL/6 mice (eight weeks, 22–25 g) were killed by cervical dislocation. All procedures were carried out in an aseptic environment. All animal procedures and experimental protocols were conducted in accordance with the guidelines approved by the Institutional Review and Ethics Board of Dongguan Tungwah Hospital, and animal care was approved by the Institutional Review and Ethics Board of Dongguan Tungwah Hospital (DHLL20161030). The study was reported following the ARRIVE guidelines and in compliance with the Public Health Service (PHS) Policy on Humane Care and Use of Laboratory Animals (policy). At the conclusion of the study, 0.3% pentobarbital sodium was used for anesthesia (concentration of 50 µg/g) through intraperitoneal injection, and the mice were euthanized by cervical dislocation.

### Immunohistochemistry

To analyze the expression of hub genes and immune cell infiltration, dewaxed tissue slices were used for immunohistochemical staining. Tissue sections were permeabilized using 0.1% Triton X-100, and 10% goat serum was used to block them for 1 h at room temperature. Tissue sections were then incubated with c-FOS antibody (1:400, ab222699, Abcam, USA), IL18 antibody (1:1000, ab223293, Abcam, USA), CD4 antibody (1:1000, ab193685, Abcam, USA), LY6G antibody (1:1000, ab238132, Abcam, USA).

### Statistical analysis

Statistical analysis was performed using the rgpubr package of R software (version 4.2.2). The boxplots were generated by the ggplot2 package of R software. Mean values of quantitative variables were evaluated by Student’s t-test, or Mann–Whitney U-test when Student’s t-test were not satisfied. For categorical variables, differences between cases and controls were analyzed by chi square (χ2) test or Fisher’s exact test. ROC analysis was conducted and ROC curves were prepared using ROC program of the R software. *p* < 0.05 was considerd as statistically significant.

## Results

### Identification of dysregulated expressed genes

**Fig. 1:**
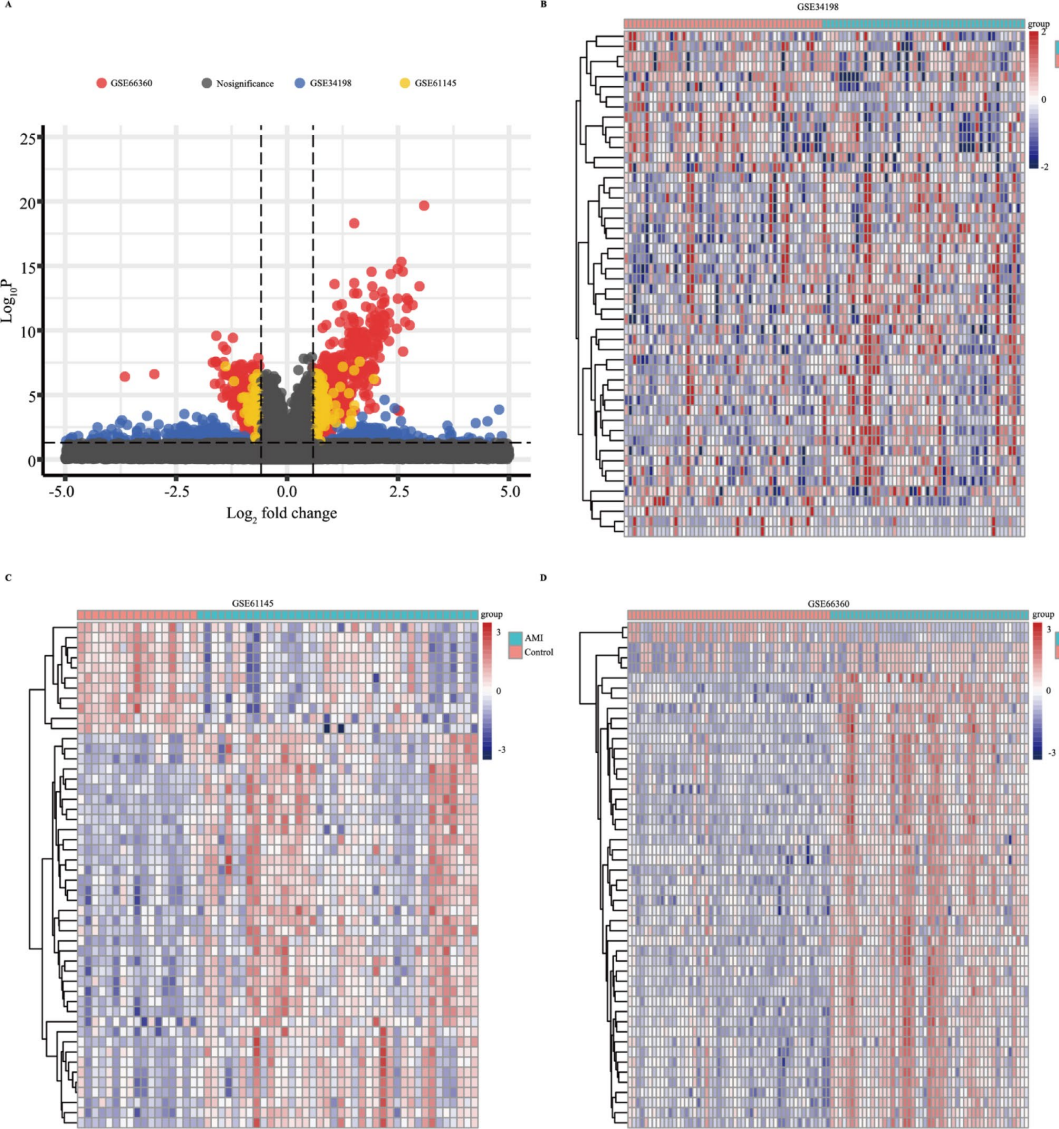
The dysregulated expressed genes in Gene Expression Omnibus (GEO) datasets. (**A**) Volcano plot of significant dysregulated expressed genes between AMI and healthy control samples. (**B**–**D**) Heatmap of the top 50 significantly upregulated or downregulated genes in GSE34198 (**B**), GSE61145 (**C**), and GSE66360 (**D**).

**Fig. 2:**
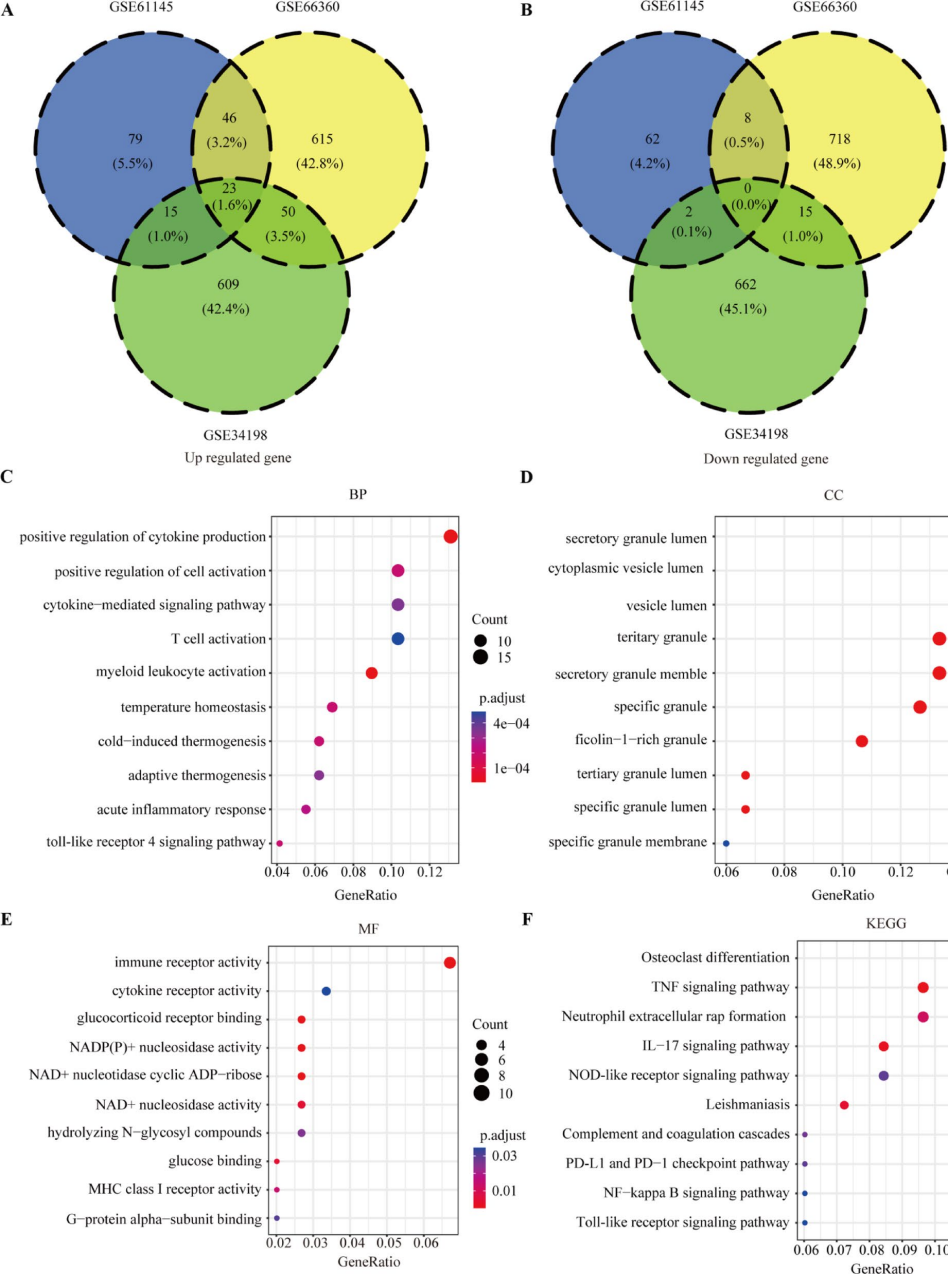
The differential expressed genes and function enrichment analysis. (**A**, **B**) 134 differentially upregulated genes (**A**) and 25 differentially downregulated genes (**B**) overlapped by GSE34198, GSE61145, and GSE66360. (**C**–**E**) The top 10 enrichment score values for significantly enriched Gene Ontology (GO) terms including biological process (**C**), cellular component (**D**), and molecular function (**E**). (**F**) Kyoto encyclopedia of genes and genomes (KEGG) analysis of differential expressed genes (DEGs)^20–22^.

As the work flow shown in Fig. S1, three GEO data sets (GSE61145, GSE34198, and GSE66360) were included in this study. A total of 697, 163, and 734 up-regulated and 679, 72, and 741 downregulated genes were obtained in GSE34198, GSE61145, and GSE66360 respectively (Fig. 1A). According to the heatmap shown in Fig. 1B–D, the expression of top 50 differential genes in GSE34198, GSE61145, and GSE66360 datasets between healthy control and AMI groups showed a differential distribution. To identify the DEGs, the dysregulated expressed genes of GSE34198, GSE61145, and GSE66360 were overlapped, and 134 differentially upregulated and 25 differentially downregulated genes (Fig. 2A,B and Table S2) were obtained.

### Function enrichment analysis

To identify biological function of the DEGs, GO enrichment and KEGG pathway enrichment analysis were conducted. Significantly enriched biological functions are summarized in Table S3. Within the biological process category, positive regulation of cytokine production, positive regulation of cell activation, cytokine-mediated signaling pathway, T cell activation, myeloid leukocyte activation, and acute inflammatory response were significantly annotated (Fig. 2C). Within the cellular component category, secretory granule lumen, cytoplasmic vesicle, vesicle lumen, and tertiary granule lumen were significantly annotated (Fig. 2D). Within the molecular function category, pathways involved in immune receptor activity, cytokine receptor activity, NAD^+^ nucleosidase activity, and MHC class I receptor activity were significantly annotated (Fig. 2E). KEGG analysis indicated that the tumor necrosis factor signaling pathway, neutrophil extracellular rap formation, interleukin (IL)-17 signaling pathway, programmed cell death ligand 1 and programmed death cell protein 1 checkpoint pathway, nuclear factor kappa beta signaling pathway, and Toll-like receptor signaling pathway were significantly enriched (Fig. 2F). These results revealed that the DEGs were involved in immune regulation during the occurrence and progression of AMI.

### Identification of hub genes by WGCNA

**Fig. 3:**
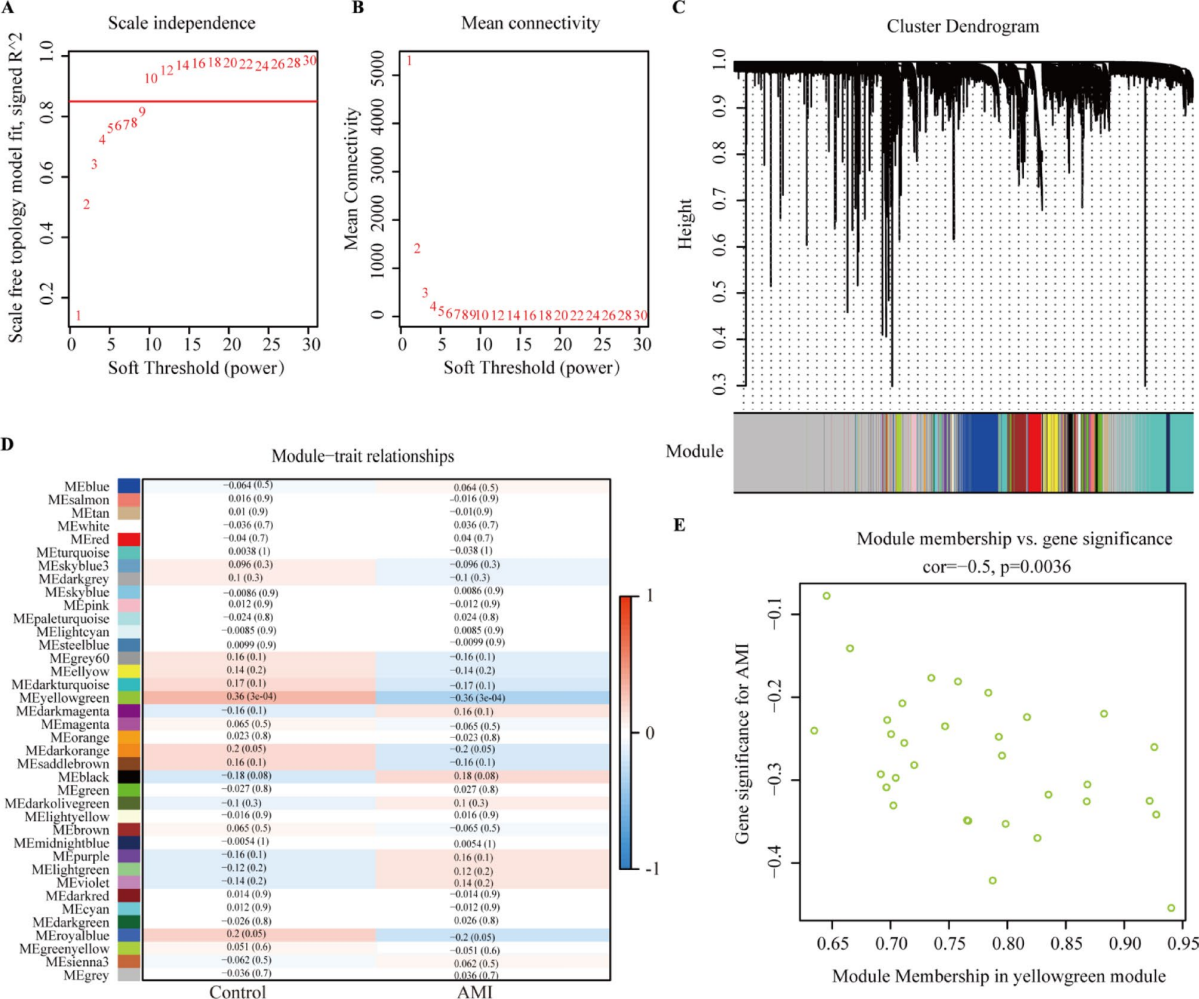
Identification of the hub genes by weighted gene co-expression network analysis (WGCNA) analysis. (**A**) Scale-free index for soft-threshold power (β) in AMI. (**B**) Mean connectivity analysis for various soft-threshold powers. (**C**) mRNA clustering dendrogram obtained by hierarchical clustering of topological overlap matrix (TOM)-based dissimilarity. (**D**) Heatmap of the correlation between module eigengene between AMI and healthy controls. (**E**) Correlation analysis between gene significance of AMI and module membership in the yellowgreen module.

Based on the expression of GEO data sets, WGCNA was performed to screen the key module and genes that mostly correlated with the AMI. To establish a scale-free network, the scale-free index and mean connectivity were calculated (Fig. 3A,B). Then, a soft threshold of 10 was implemented. MEyellowgreen module showed the strongest correlation with AMI features (*r* = -0.36; Fig. 3C,D). Scatter diagrams were constructed for correlation analysis between gene significance for AMI and module membership in the yellowgreen module, which revealed that genes were significantly corelated with AMI (corelation = -0.5, *p* = 0.0036; Fig. 3E).

### Identification of hub genes by machine learning

**Fig. 4:**
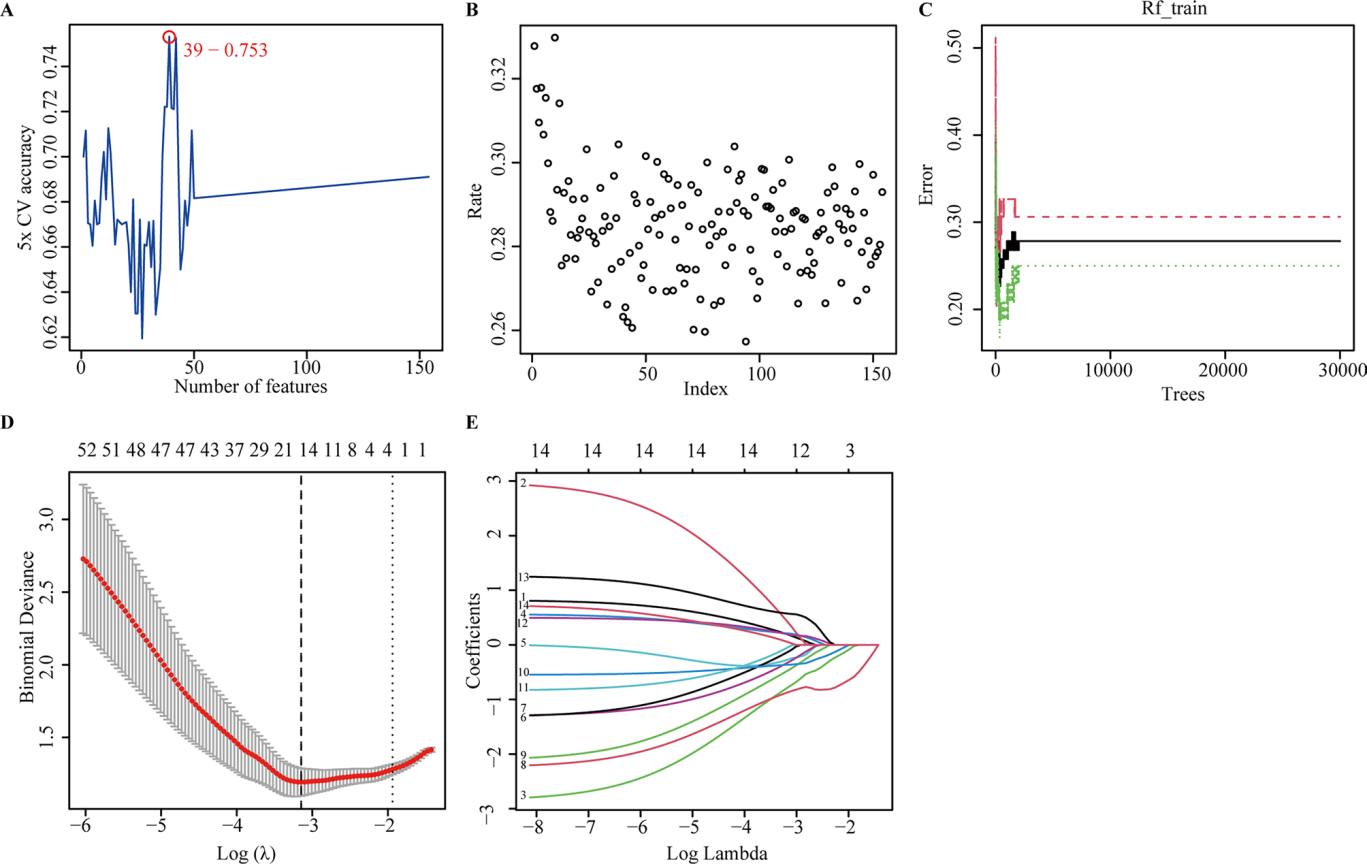
Identification of the hub genes by machine learning. (**A**) support vector machine (SVM) analysis. (**B**, **C**) The index (**B**) and number (**C**) grown for random forest (RF) analysis; (**D**, **E**) Cross-validation to select the optimal tuning parameter log (Lambda) (**D**) and least absolute shrinkage and selection operator (LASSO) coefficient profiles (**E**) by LASSO regression analysis.

**Fig. 5:**
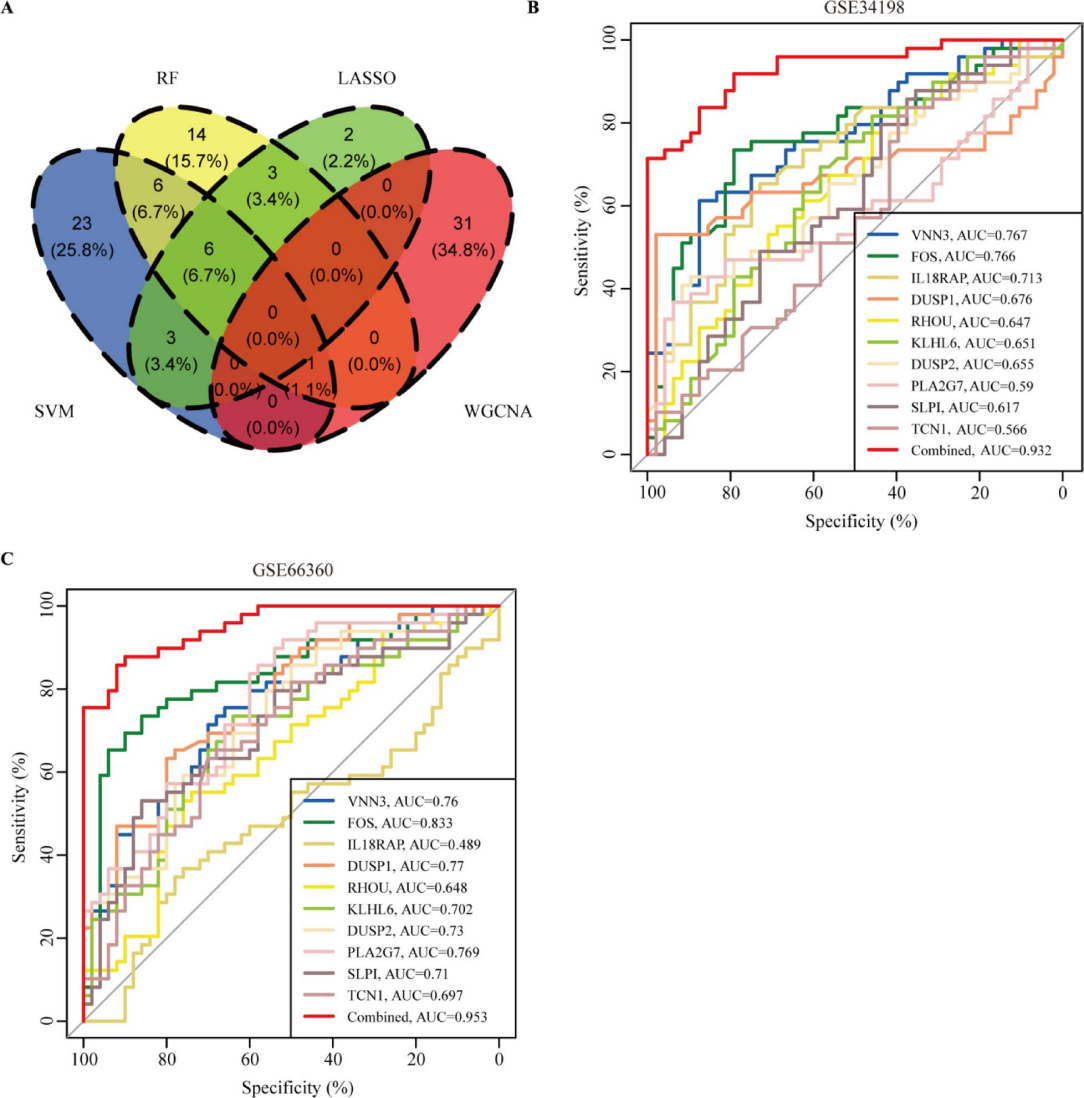
Development and validation of the diagnostic model. (**A**) The intersection of candicated genes using the weighted gene co-expression network analysis (WGCNA), support vector machine (SVM), random forest (RF) and least absolute shrinkage and selection operator (LASSO) analysis. (**B**) The clinical diagnostic model in GSE34198. (**C**) The clinical diagnostic model in GSE66360.

To further reveal the hub genes, the 159 DEGs were screened by SVM in GSE34198. Results indicated that 39 hub genes were identified with an accuracy of 0.753 (Fig. 4A and Table S2, S4). These DEGs were further screened by RF; after determining mtry and ntree parameters, 30 stable genes were retained by RF analysis, which were ranked as important factors in the division of AMI (Fig. 4B,C and Table S2). LASSO analysis identified that 14 hub genes were clarified (Fig. 4D,E and Table S2). The hub genes screened by SVM, RF, LASSO and WGCNA were further overlapped, and 19 hub genes were obtained (Fig. 5A). The stepwise regression method was used to further reduce the gene set, and finally 10 hub genes, including *VNN3*,* FOS*,* IL18RAP*,* DUSP1*,* RHOU*,* KLHL6*,* DUSP2*,* PLA2G7*,* SLPI*, and *TCN1* were identified.

### Development and validation of diagnostic model

*DUSP1*,* VNN3*, and *FOS* showed a positive relationship, while *RHOU* displayed a negative relationship with *DUSP1*,* VNN3*, and *FOS* (Fig. S2). Expression analysis indicated that the 10 hub genes were dysregulated in GSE34198, GSE66360 and GSE66360 (Fig. S3A–C). However, only four hub genes, including *VNN3*,* FOS*,* IL18RAP*, and *DUSP1*, were identified in GSE61145. Therefore, GSE34198 and GSE66360 were selected to develop and validate the diagnostic model. We first assessed the diagnostic values of the 10 hub genes in GSE34198 and GSE66360 by ROC curves. The diagnostic values of *VNN3*,* FOS*,* IL18RAP*,* DUSP1*,* RHOU*,* KLHL6*,* DUSP2*,* PLA2G7*,* SLPI*, and *TCN1* in GSE34198 and GSE66360 were 0.767 vs. 0.76, 0.766 vs. 0.833, 0.713 vs. 0.489, 0.676 vs. 0.77, 0.647 vs. 0.648, 0.651 vs. 0.702, 0.655 vs. 0.73, 0.59 vs. 0.769, 0.617 vs. 0.71, and 0.566 vs. 0.697, respectively (Fig. 5B,C). Nevertheless, We found that the combine model of 10 hub genes was 0.932 vs. 0.953. These results indicated that this combined model can act as a diagnostic marker to predict AMI and may indicate the involvement of immune cell infiltration during AMI development.

### Immune cell infiltration and correlation analysis

To explore the role of immune cells in AMI, principal component analysis (PCA) was conducted according to the expression profile of the 10 hub genes, which efficiently distinguished AMI and healthy control samples (Fig. S4A and Table S5). CIBERSORT method was applied to analyze the infiltration of 22 types of immune cells in clinical samples. The correlation analysis revealed negative regulation among immune cells, such as between resting NK cells and gamma delta T cells, naïve CD4 T cells and M0 macrophages, naive CD4 T cells and neutrophils, activated CD4 memory T cells and Tregs, and neutrophils and CD8 T cells (Fig. S4B). The AMI group showed a higher fraction of naive B cells and activated CD4 memory T cells and a lower fraction of resting mast cells than did the healthy control group (*p* < 0.05; Fig. S4C).

**Fig. 6:**
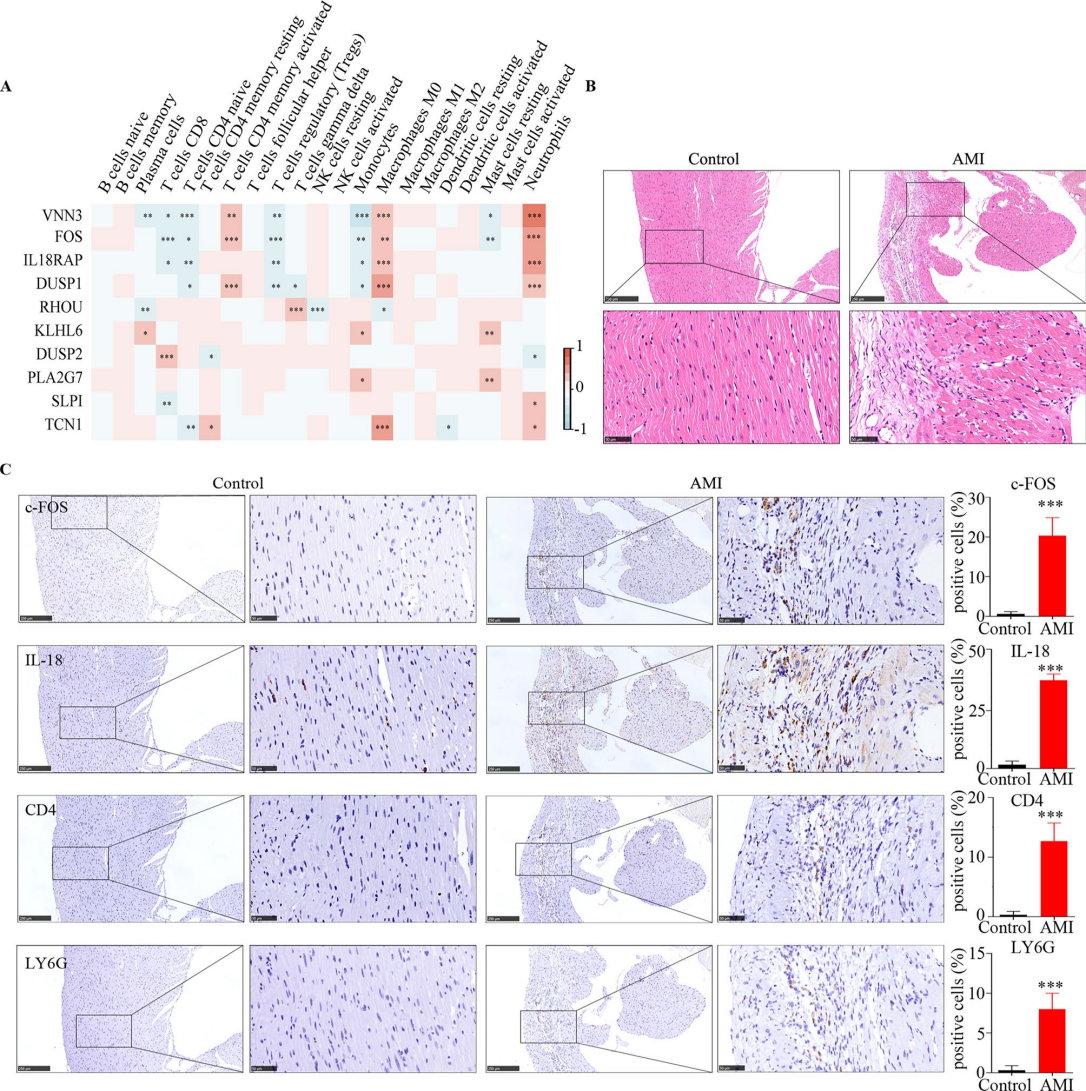
Immune cell infiltration in AMI. (**A**) Heatmap of correlations between the 10 hub genes and infiltrated immune cells. Immunohistochemical staining. (**B**) Hematoxylin-eosin staining of control and AMI in *vivo*. (**C**) The expression of c-FOS, IL18RAP, CD4 and LY6G by immunohistochemistry. *** means *p* < 0.001.

The expressions of *VNN3*,* FOS*,* IL18RAP*, and *DUSP1* were positively correlated with activated CD4 memory T cells, M0 macrophages, and neutrophils and negatively correlated with CD8 T cells, naive CD4 T cells, Tregs, monocytes, and resting mast cells. *RHOU* in Tregs, *KLHL6* in plasma cells, monocytes, and resting mast cells, *DUSP2* in CD8 T cells, *PLA2G7* in monocytes and resting mast cells, *SLPI* in neutrophils, and *TCN1* in resting CD4 memory T cells, M0 macrophages, and neutrophils were positively correlated. However, *RHOU* in plasma cells, resting NK cells, and M0 macrophages, *DUSP2* in naïve CD4 T cells and neutrophils, and *TCN1* in CD4 naive T cells and resting dendritic cells were negatively correlated (*p* < 0.05; Fig. 6A). As shown in Fig. 6B, HE staining showed that myocardial infarction tissue showed massive congestion and edema with tissue necrosis, which indicated an acute myocardial infarction model were successfully established. Immunohistochemical staining of continuous tissue sections were used to detect the protein expression of hub genes (*FOS* and *IL18RAP*), and immune cells. The levels of FOS, IL18RAP, CD4 naive T (CD4), and neutrophils (LY6G) were significantly upregulated in AMI (Fig. 6C). Together, these results suggest that the 10 hub genes that regulate immune cell infiltration may be potential diagnostic biomarkers for AMI.

## Discussion

Recent studies have revealed that 17 million people suffer from cardiovascular disease, and nearly half of all cardiovascular deaths are associated with AMI^23^. Despite the improvements in clinical treatment over the past decades, AMI remains a leading cause of mortality and hospitalization globally, mostly in middle-aged patients^24^. However, owing to the complexity and heterogeneity, AMI is considered as a progressively and inevitably pathological process^25^. Clinically, AMI could be only detected at a reversible stage, and clinical biomarkers are not precise enough to evaluate the risk of AMI. Therefore, new biomarkers for an early-stage detection of AMI need to be further explored.

In this study, three datasets were downloaded from the GEO database, and a total of 159 dysregulated expressed genes, including 134 upregulated and 25 downregulated genes, were identified. WGCNA and machine learning techniques found significant differences in the expression of *VNN3*,* FOS*,* IL18RAP*,* DUSP1*,* RHOU*,* KLHL6*,* DUSP2*,* PLA2G7*,* SLPI*, and *TCN1*, which were employed to develop diagnostic models to predict the risk of AMI. According to our results, these ten hub genes displayed ideal predictive performance for AMI, as assessed by the ROC curve. VNN3, a secreted protein, is induced by oxidative stress^26^, and VVN2 can regulate transmigration of activated neutrophils. FOS has been reported to be involved in the occurrence of AMI^27^. The genes of the IL18 family, including *IL18RAP*, are involved in developing coronary atherosclerosis and are associated with pathological development and the risk of acute coronary events^28^. DUSP1 overexpression potently inhibits apoptosis of the cardiomyocytes associated with ischemia/reperfusion injury mediated by TRIM11^29^. RHOU, one of RHO GTPase family members, regulates the polarity and architecture of cells^30^. KLHL6 deficiency impairs transitional B cell survival and differentiation^31^. The decrease in PLA2G7 levels may mediate the immune-metabolic effects of caloric restriction and could lower inflammatory response^32^. SLPI can inhibit the release of leukocyte elastase, neutrophil elastase, and mast cell chymase^33^. TCN1 acts as a potential prognostic biomarker and correlates with immune infiltrates in lung adenocarcinoma^34^. At last, through the *vivo* experiments validation of our results, the upregulated expression patterns of *FOS* and *IL18RAP* were associated with immunce cell infiltration in AMI. These results indicate that the hub genes are associated with inflammation and immune response. However, the function and immune mechanism of the hub genes in AMI needs further exploration.

Based on the expression of DEGs, GO and KEGG pathways were mainly distributed in inflammation- and immunity-related pathways. Immune cells are involved in the occurrence and development of AMI, which lead to atherosclerosis, while they prevent the lesion^35^. Myocardial infarction is associated with the destruction of immune and inflammatory balance; however, the immune cells that are activated in the process of AMI have not been specified. Immune cell infiltration analysis revealed that naïve B cells and activated CD4 memory T cells CD4 were upregulated, while a low fraction of resting mast cells was downregulated in AMI. Correlation analysis identified that the hub genes were involved in regulating the immune balance. Immunohistochemical experiments showed significant upregulation of CD4 T cells and neutrophils in AMI. The dysregulation of immune and inflammatory responses is significantly associated with AMI. Therefore, targeting the hub genes may be a promising way to treat AMI.

This study has a few limitations. First, this was a retrospective study based on public datasets, and there may be some causal inference and selection bias. Second, the diagnostic model was constructed based on the GSE34198 and GSE66360 datasets, the diagnostic and prognostic values of which need to be further validated prior to consideration of widespread use. Third, the roles and mechanism of hub genes in immune cell infiltration and inflammatory responses need to be further explored. Finally, our study mainly extracted the clinical data of AMI patients, which may ignore the dynamic changes of coronary artery disease with time.

## Conclusion

In summary, 10 hub genes (*VNN3*,* FOS*,* IL18RAP*,* DUSP1*,* RHOU*,* KLHL6*,* DUSP2*,* PLA2G7*,* SLPI*, and *TCN1*) were identified as diagnostic biomarkers for AMI. The dysregulated immune cells mediated by the hub genes, such as, naïve B cells, activated CD4 memory T cells, and mast cells, may be associated with the progression of AMI. Targeting the dysregulated expressed genes may provide useful diagnostic biomarkers to formulate effective treatment strategies for AMI.

## Electronic supplementary material

Below is the link to the electronic supplementary material.

Supplementary Material 1

Supplementary Material 2

Supplementary Material 3

Supplementary Material 4

Supplementary Material 5

Supplementary Material 6

## Data Availability

All data used are contained within the manuscript; these data were obtained from gene expression omnibus database (GEO, https://www.ncbi.nlm.nih.gov/geo/). The datasets used and/or analysed during the current study available from the corresponding author on reasonable request.

## Abbreviations

AMI: Acute myocardial infarction
cTns: Cardiac troponins
GEO: Gene expression omnibus
DEGs: Differential expressed genes
GO: Gene ontology
KEGG: Kyoto encyclopedia of genes and genomes
SVM: Support vector machine
RF: Random forest
LASSO: Least absolute shrinkage and selection operator
WGCNA: Weighted gene co-correlation network analysis
ROC: Receiver operating characteristic
PCA: Principal component analysis
BP: Biological process
CC: Cellular component
MF: Molecular function
