# Supplementary material for: Identification of clinical diagnostic and immune cell infiltration characteristics of acute myocardial infarction with machine learning approach
Source: Sci Rep. 2025 Jul 20;15:26315. doi: 10.1038/s41598-025-11957-0 (PMC12277416; doi:10.1038/s41598-025-11957-0)
Supplement: Supplementary file 6 — Supplementary Material 6 [file 41598_2025_11957_MOESM6_ESM.docx]

**Supplementary Materials**

**
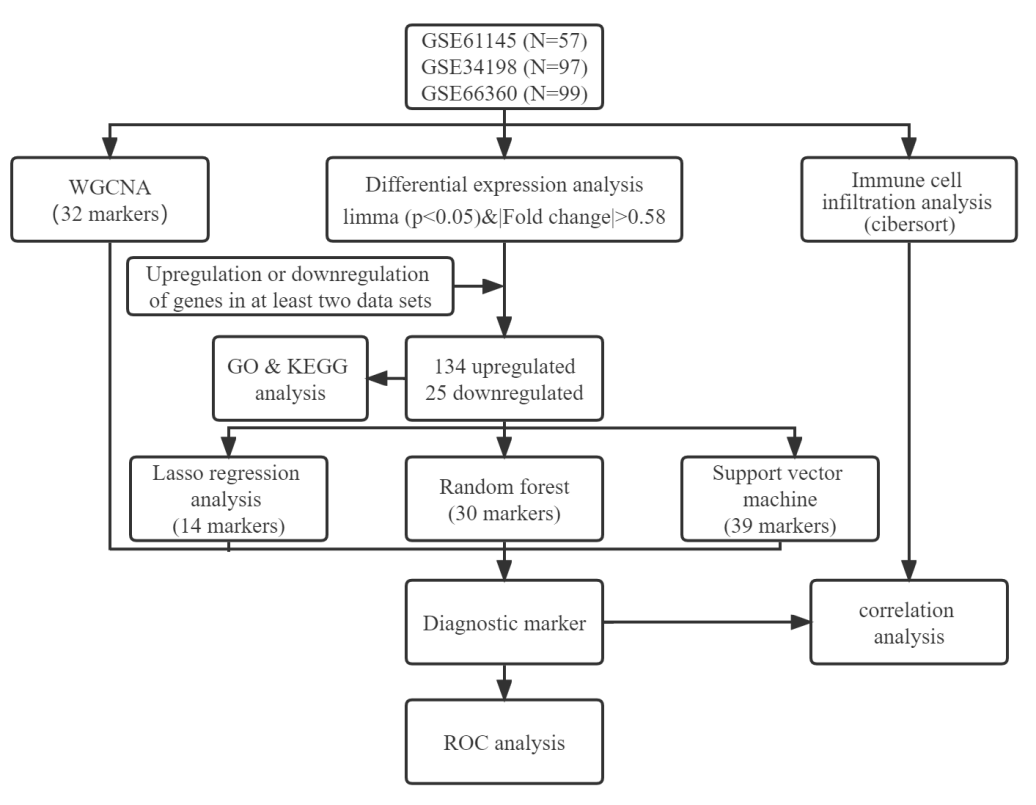
**

**Figure S1.** The workflow in this study.

**
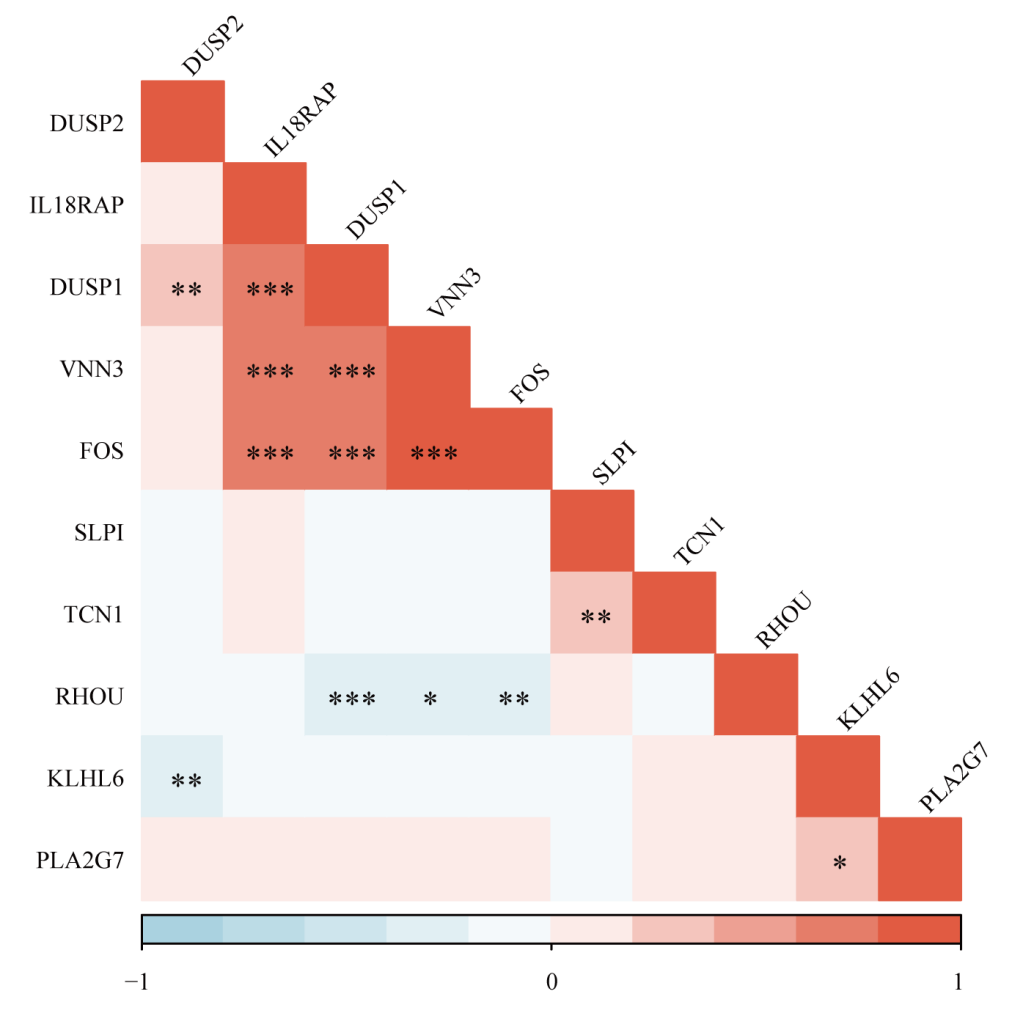
**

**Figure S2.** Correlation analysis of the 10 hub genes.

**
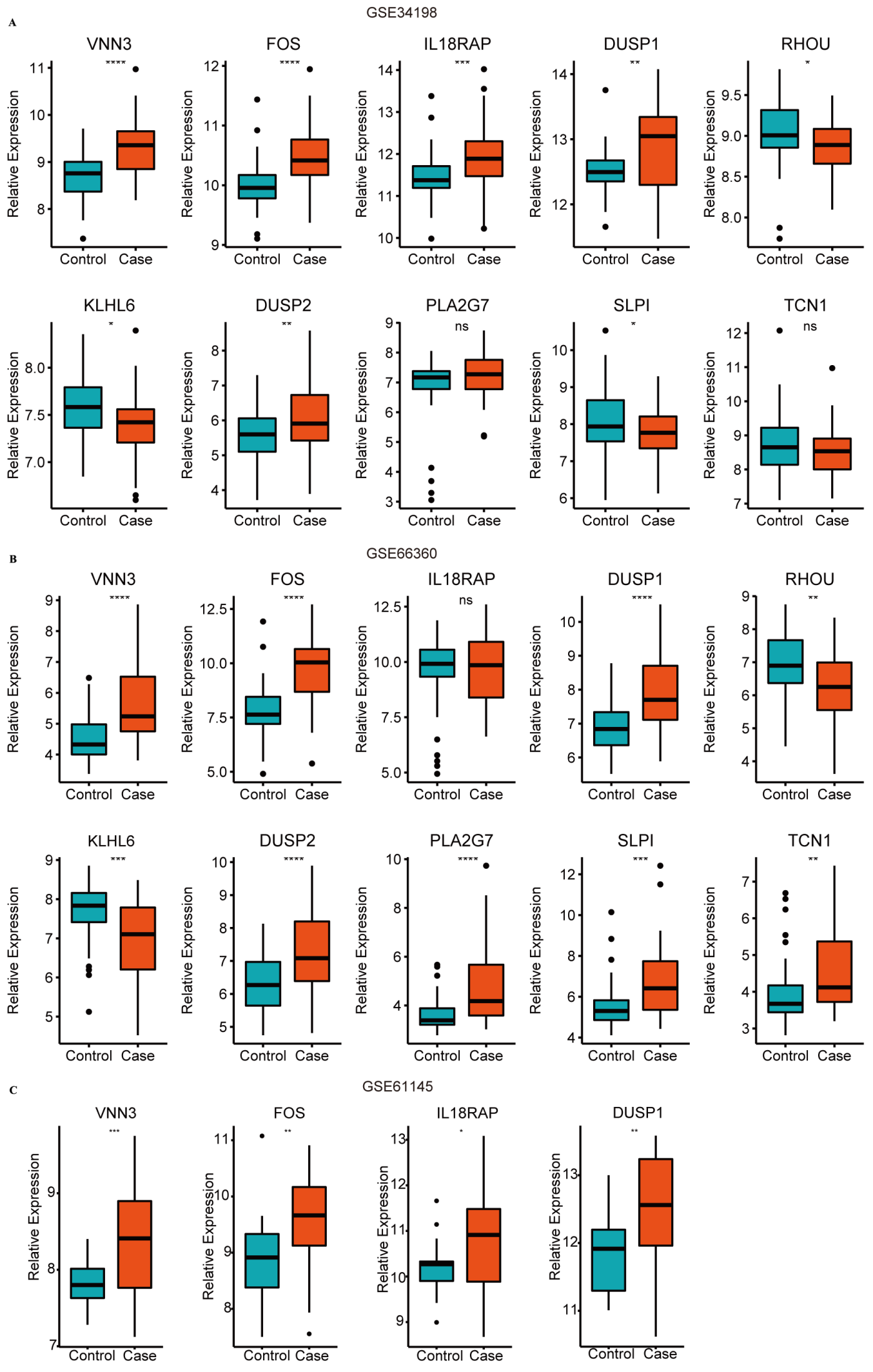
**

**Figure S3.** mRNA expression of the 10 hub genes in GSE34198 (**A**), GSE66360 (**B**), and GSE61145 (**C**).

**
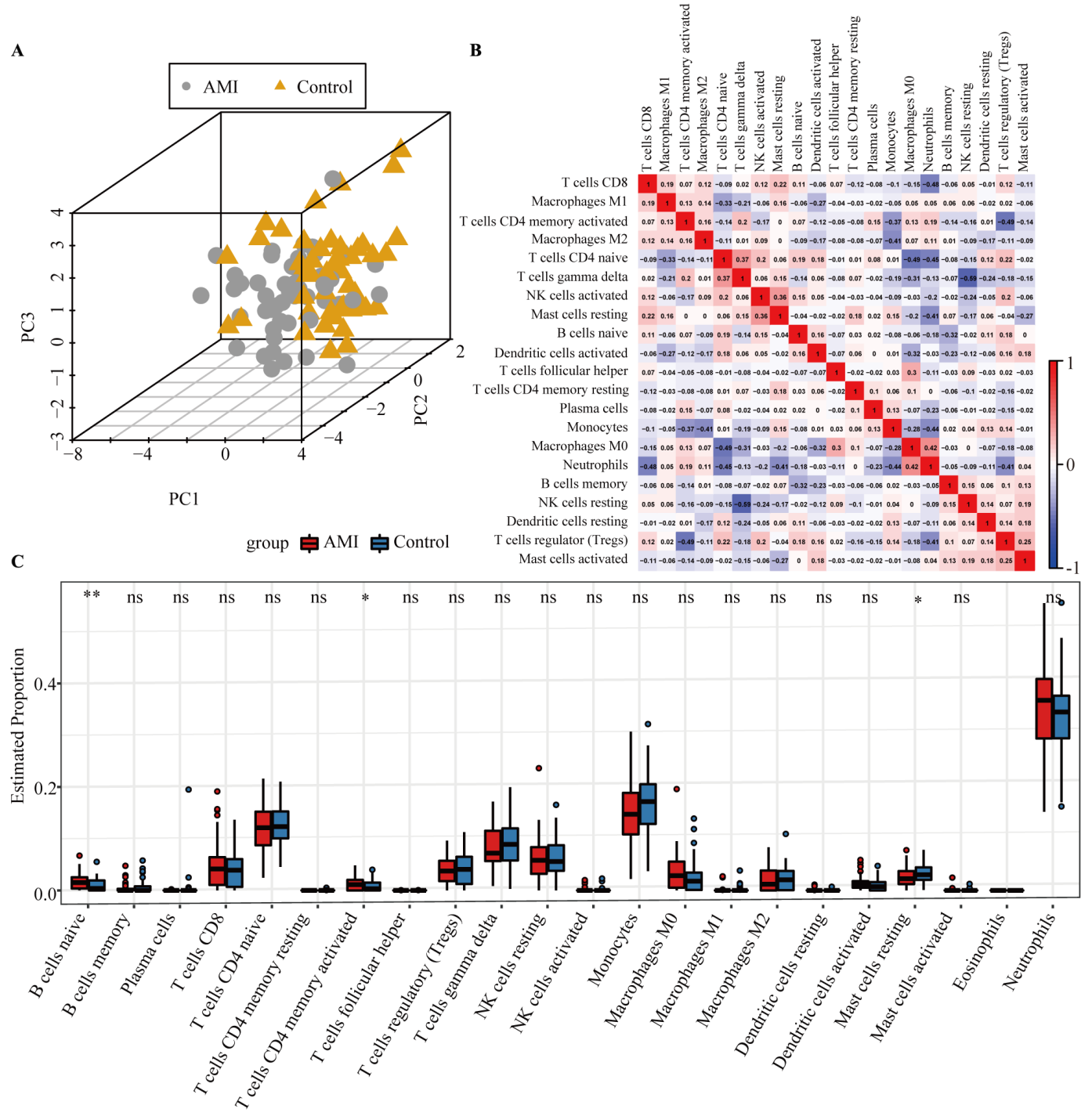
**

**Figure S4.** Correlation analysis among immune cells. (**A**) principal component analysis (PCA) between AMI and healthy control samples. (**B**) Correlation analysis among fractions of 22 types of immune cells. Red represents positive correlation, white represents the same correlation levels, and blue represents negative correlation. (**C**) The difference of immune cell infiltration between AMI and healthy control samples.

**Table S1.** The clinical information of GSE34198, GSE66360, and GSE61145.

**Table S2.** The dysregulated expressed genes by limma, Support Vector Machine (SVM), Random Forest (RF), Least Absolute Shrinkage and Selection Operator (LASSO), and Weighted Gene Co-expression Network Analysis (WGCNA) analyses.

**Table S3.** Gene ontology (GO) annotation and Kyoto Encyclopedia of Genes and Genomes (KEGG) pathway enrichment analysis.

**Table S4.** The accuracy and error of support vector machine (SVM) analysis.

**Table S5.** Immune cell infiltration analysis by CIBERSORT.
